# Fixed Point Results for Generalized Chatterjea Type Contractive Conditions in Partially Ordered *G*-Metric Spaces

**DOI:** 10.1155/2014/341751

**Published:** 2014-02-11

**Authors:** Safeer Hussain Khan, Mujahid Abbas, Talat Nazir

**Affiliations:** ^1^Department of Mathematics, Statistics and Physics, Qatar University, Doha 2713, Qatar; ^2^Department of Mathematics and Applied Mathematics, University of Pretoria, Lynnwood Road, Pretoria 0002, South Africa; ^3^COMSATS Institute of Information Technology, Abbotabad, Pakistan

## Abstract

In the framework of ordered *G*-metric spaces, fixed points of maps that satisfy the generalized (*ψ*, *φ*)-Chatterjea type contractive conditions are obtained. The results presented in the paper generalize and extend several well known comparable results in the literature.

## 1. Introduction and Preliminaries

The study of fixed points of mappings satisfying certain contractive conditions has been at the center of rigorous research activity. Mustafa and Sims [1] generalized the concept of a metric space. Based on the notion of generalized metric spaces, Mustafa et al. [1–5] obtained some fixed point theorems for mappings satisfying different contractive conditions. Abbas and Rhoades [6] initiated the study of a common fixed point theory in generalized metric spaces. Abbas et al. [7] and Chugh et al. [8] obtained some fixed point results for maps satisfying property *P* in *G*-metric spaces. Recently, Shatanawi [9] proved some fixed point results for self mappings in a complete  *G*-metric space under some contractive conditions related to a nondecreasing map *ϕ* : *R* ^+^ → *R* ^+^ with lim⁡_*n*→*∞*_⁡*ϕ* ^*n*^(*t*) = 0 for all *t* ≥ 0. Recently, Saadati et al. [10] proved some fixed point results for contractive mappings in partially ordered *G*-metric spaces.

Ran and Reurings [11] extended Banach contraction principle in partially ordered metric spaces with some applications to linear and nonlinear matrix equations, while Nieto and Rodríguez-López [12] extended the result of Ran and Reurings and applied their main result to obtain a unique solution for a first order ordinary differential equation with periodic boundary conditions. Bhaskar and Lakshmikantham [13] introduced the concept of mixed monotone mappings and obtained some coupled fixed point results. Also, they applied their results to a first order differential equation with periodic boundary conditions.

Alber and Guerre-Delabriere [14] introduced the concept of weakly contractive mappings and proved the existence of fixed points of such mappings in Hilbert spaces. Thereafter, in 2001, Rhoades [15] proved the fixed point theorem which is one of the generalizations of Banach's contraction mapping principle. Weakly contractive mappings are closely related to the mappings of Boyd and Wong [16] and of Reich types [17]. Recently, Dorić [18] proved a common fixed point theorem for generalized (*ψ*, *ϕ*)-weakly contractive mappings. Fixed point problems involving weak contractions and mappings satisfying weak contractive type inequalities have been studied by many authors (see [8, 14, 15, 18–21] and references cited therein).

In this paper, we generalize the Chatterjea type contraction mappings to generalized (*ψ*, *φ*)-Chatterjea type contraction mappings and derive some fixed point results for single-valued mappings in ordered generalized metric spaces.

Consistent with Mustafa and Sims [1], the following definitions and results will be needed in the sequel.

**Definition 1**

Let  *X*  be a nonempty set. Suppose that a mapping  *G* : *X* × *X* × *X* → *R* ^+^  satisfies

(G_1_) *G*(*x*, *y*, *z*) = 0 if *x* = *y* = *z*;
(G_2_) 0 < *G*(*x*, *y*, *z*) for all *x*, *y*, *z* ∈ *X*, with *x* ≠ *y*;
(G_3_) *G*(*x*, *x*, *y*) ≤ *G*(*x*, *y*, *z*) for all *x*, *y*, *z* ∈ *X*, with *y* ≠ *z*;
(G_4_) *G*(*x*, *y*, *z*) = *G*(*x*, *z*, *y*) = *G*(*y*, *z*, *x*) = ⋯ (symmetry in all three variables);
(G_5_) *G*(*x*, *y*, *z*) ≤ *G*(*x*, *a*, *a*) + *G*(*a*, *y*, *z*) for all *x*, *y*, *z*, *a* ∈ *X*.

Then, *G* is called a *G*-metric on *X* and (*X*, *G*) is called a *G*-metric space.

**Definition 2**

A sequence  {*x*_*n*_}  in a  *G*-metric space  *X*  is

- a  *G*-*Cauchy* sequence if, for every *ε* > 0, there is a natural number *n*_0_ such that for all *n*, *m*, *l* ≥ *n*_0_, *G*(*x*_*n*_, *x*_*m*_, *x*_*l*_) < *ε*,
- a  *G*-*Convergent* sequence if, for any *ε* > 0, there is an *x* ∈ *X* and an *n*_0_ ∈ *ℕ*, such that for all *n*, *m* ≥ *n*_0_, *G*(*x*_*n*_, *x*_*m*_, *x*) < *ε*.

A *G*-metric space on  *X*  is said to be  *G*-complete if every  *G*-Cauchy sequence in  *X*  is  *G*-convergent in  *X*. It is known that {*x*_*n*_}  *G*-converges to *x* ∈ *X* if and only if *G*(*x*_*m*_, *x*_*n*_, *x*) → 0 as *n*, *m* → *∞*.

**Proposition 3 (see [1])**

*Let X* *be a G* *-metric space. Then, the following are equivalent.*

1. *The sequence *{*x*_*n*_}* is G-convergent to x*.
2. *G*(*x*_*n*_, *x*_*n*_, *x*) → 0* as n* → *∞*.
3. *G*(*x*_*n*_, *x*, *x*) → 0* as n* → *∞*.
4. *G*(*x*_*n*_, *x*_*m*_, *x*) → 0* as n*, *m* → *∞*.

**Proposition 4 (see [1])**

*Let X* *be a G* *-metric space. Then, the following are equivalent.*

1. *The sequence *{*x*_*n*_}* is G-Cauchy.*
2. *For every ε* > 0*, there exists n*_0_ ∈ *ℕ, such that for all n*, *m* ≥ *n*_0_, *G*(*x*_*n*_, *x*_*m*_, *x*_*m*_) < *ε; that is, if G*(*x*_*n*_, *x*_*m*_, *x*_*m*_) → 0* as n*, *m* → *∞*.

**Definition 5**

A *G*-metric on  *X*  is said to be symmetric if *G*(*x*, *y*, *y*) = *G*(*y*, *x*, *x*) for all *x*, *y* ∈ *X*.

**Proposition 6**

Every *G*-metric on *X* defines a metric  *d*_*G*_  on  *X*  by

(1) $$\begin{array}{c} d_{G}\left(x,y\right)=G\left(x,y,y\right)+G\left(y,x,x\right),\;\forall x,y\in X. \end{array}$$

For a symmetric *G*-metric space, one obtains

(2) $$\begin{array}{c} d_{G}\left(x,y\right)=2G\left(x,y,y\right),\;\forall x,y\in X. \end{array}$$

However, if  *G*  is not symmetric, then the following inequality holds:

(3) $$\begin{array}{c} \frac{3}{2}G\left(x,y,y\right)\leq d_{G}\left(x,y\right)\leq 3G\left(x,y,y\right),\;\forall x,y\in X. \end{array}$$

First, we recall some basic definitions and notations.

Let (*X*, *d*) be a metric space. A map *T* : *X* → *X* is said to be

- *Kannan type* (see [22]) if there exists a *k* ∈ (0, 1/2] such that  *d*(*Tx*, *Ty*) ≤ *k*[*d*(*x*, *Tx*) + *d*(*y*, *Ty*)]  for all  *x*, *y* ∈ *X*;
- *Chatterjea type* [20] if there exists a *k* ∈ (0, 1/2] such that *d*(*Tx*, *Ty*) ≤ *k*[*d*(*x*, *Ty*) + *d*(*y*, *Tx*)] for all *x*, *y* ∈ *X*.

**Definition 7**

We define two classes of mappings as follows:

 Ψ = {*ψ* | *ψ* : [0, *∞*)→[0, *∞*) is continuous and nondecreasing with *ψ*(*t*) = 0  if and only if *t* = 0} and
 Φ = {*φ* | *φ* : [0, *∞*)^5^ → [0, *∞*) is lower semi-continuous with *φ*(*t*_1_, *t*_2_, *t*_3_, *t*_4_, *t*_5_) = 0 if and only if *t*_1_ = *t*_2_ = *t*_3_ = *t*_4_ = *t*_5_ = 0}.

**Definition 8**

An ordered partial *G*-metric space is said to have a sequential limit comparison property if for every nondecreasing sequence (nonincreasing sequence) {*x*_*n*_} in  *X*  such that *G*(*x*_*n*_, *x*, *x*) → 0 as *n* → *∞* implies that *x*_*n*_⪯*x*  (*x*⪯*x*_*n*_), respectively.

## 2. Fixed Point Results

In this section, we obtain fixed point results for mappings satisfying generalized  (*φ*, *ψ*)-Chatterjea type contractive conditions on partially ordered complete generalized metric space. We start with the following result.

**Theorem 9**

Let (*X*, ⪯) be a partially ordered set and *f* be a nondecreasing self mapping on a complete *G*-metric space *X* satisfying

(4) $$\begin{array}{c} \psi \left(G\left(fx,fy,fy\right)\right)\leq \psi \left(M\left(x,y,y\right)\right)-\varphi \left(N\left(x,y,y\right)\right), \end{array}$$

where *ψ* ∈ Ψ, *φ* ∈ Φ with

(5) M(x,y,y)=max⁡{G(x,y,y),G(x,fx,fx),G(y,fy,fy),[G(x,fy,fy)+G(y,fx,fx)]2},N(x,y,y)=(G(x,y,y),G(x,fx,fx),G(y,fy,fy),G(x,fy,fy),G(y,fx,fx))

for all *x*, *y* ∈ *X* with *x*⪯*y*. Suppose that there exists *x*_0_ ∈ *X* with *x*_0_⪯*fx*_0_. If *f* is continuous or *X* a sequential limit comparison property, then *f* has a fixed point in *X*.

**Proof**

If *fx*_0_ = *x*_0_, there is nothing to prove. Suppose that *fx*_0_ ≠ *x*_0_. Since *x*_0_⪯*fx*_0_ and *f* is nondecreasing, we have

(6) $$\begin{array}{c} x_{0}⪯fx_{0}⪯f^{2}x_{0}⪯\cdots ⪯f^{n}x_{0}⪯\cdots . \end{array}$$

Define a sequence  {*x*_*n*_}  by *x*_*n*_ = *f* ^*n*^ *x*_0_ so that *x*_*n*+1_ = *fx*_*n*_. We may assume that *G*(*x*_*n*_, *x*_*n*+1_, *x*_*n*+1_) > 0 for every  *n* ∈ *ℕ*. If not, then *x*_*n*_ = *x*_*n*+1_ for some *n* and *x*_*n*_ becomes a fixed point of *f*. Using (4), we obtain

(7) ψ(G(xn+1,xn+2,xn+2))  =ψ(G(fxn,fxn+1,fxn+1))  ≤ψ(M(xn,xn+1,xn+1))−φ(N(xn,xn+1,xn+1)),

where

(8) M(xn,xn+1,xn+1)  =max⁡{G(xn,xn+1,xn+1),G(xn,fxn,fxn),G(xn+1,fxn+1,fxn+1),[G(xn,fxn+1,fxn+1)+G(xn+1,fxn,fxn)]2}  =max⁡{G(xn,xn+1,xn+1),G(xn,xn+1,xn+1),G(xn+1,xn+2,xn+2),[G(xn,xn+2,xn+2)+G(xn+1,xn+1,xn+1)]2}  =max⁡{G(xn,xn+1,xn+1),G(xn+1,xn+2,xn+2)},N(xn,xn+1,xn+1)  =(G(xn,xn+1,xn+1),G(xn,fxn,fxn),G(xn+1,fxn+1,fxn+1),G(xn,fxn+1,fxn+1),G(xn+1,fxn,fxn))  =(G(xn,xn+1,xn+1),G(xn,xn+1,xn+1),G(xn+1,xn+2,xn+2),G(xn,xn+2,xn+2),G(xn+1,xn+1,xn+1))  =(G(xn,xn+1,xn+1),G(xn,xn+1,xn+1),G(xn+1,xn+2,xn+2),G(xn,xn+2,xn+2),0).

If we take *G*(*x*_*n*+1_, *x*_*n*+2_, *x*_*n*+2_) ≥ *G*(*x*_*n*_, *x*_*n*+1_, *x*_*n*+1_) for some *n* ≥ 0, it follows that *φ*(*N*(*x*_*n*_, *x*_*n*+1_, *x*_*n*+1_)) = 0, a contradiction. Therefore, for all *n* ≥ 0,

(9) $$\begin{array}{c} G\left(x_{n+1},x_{n+2},x_{n+2}\right)<G\left(x_{n},x_{n+1},x_{n+1}\right) \end{array}$$

so that *M*(*x*_*n*_, *x*_*n*+1_, *x*_*n*+1_) = *G*(*x*_*n*_, *x*_*n*+1_, *x*_*n*+1_). Now {*G*(*x*_*n*+1_, *x*_*n*+2_, *x*_*n*+2_)} is a decreasing sequence, so there exists *L* ≥ 0 such that lim⁡_*n*→*∞*_⁡*G*(*x*_*n*+1_, *x*_*n*+2_, *x*_*n*+2_) = *L*. This gives lim⁡_*n*→*∞*_⁡*G*(*x*_*n*_, *x*_*n*+1_, *x*_*n*+1_)  =  lim⁡_*n*→*∞*_⁡*M*(*x*_*n*_, *x*_*n*+1_, *x*_*n*+1_) = *L*. By lower semicontinuity of *φ*,

(10) $$\begin{array}{c} \varphi \left(L,L,L,L,0\right)\leq \underset{n\to \infty }{\lim\;\inf}\varphi \left(N\left(x_{n},x_{n+1},x_{n+1}\right)\right). \end{array}$$

We claim that *L* = 0. Taking the upper limits as *n* → *∞* on both sides of

(11) ψ(G(xn+1,xn+2,xn+2))  ≤ψ(M(xn,xn+1,xn+1))−φ(N(xn,xn+1,xn+1)),

we have

(12) ψ(L)≤ψ(L)−lim⁡ inf⁡n→∞φ(N(xn,xn+1,xn+1))≤ψ(L)−φ(L,L,L,L,0).

This implies *φ*(*L*, *L*, *L*, *L*, 0) = 0 and we conclude that

(13) $$\begin{array}{c} \underset{n\to \infty }{\lim}G\left(x_{n+1},x_{n+2},x_{n+2}\right)=0. \end{array}$$

Next, we show that {*x*_*n*_} is a *G*-Cauchy sequence in  *X*. If not, then there exist *ε* > 0 and integers *n*_*k*_ and *m*_*k*_ with *m*_*k*_ > *n*_*k*_ > *k* such that

(14) $$\begin{array}{c} G\left(x_{n_{k}},x_{m_{k}},x_{m_{k}}\right)\geq \varepsilon ,\;\;G\left(x_{n_{k}},x_{m_{k}-1},x_{m_{k}-1}\right)<\varepsilon . \end{array}$$

A joint effect of (13) and (14) on

(15) ε≤G(xnk,xmk,xmk)≤G(xnk,xmk−1,xmk−1)+G(xmk−1,xmk,xmk)

yields

(16) $$\begin{array}{c} \underset{k\to \infty }{\lim}G\left(x_{n_{k}},x_{m_{k}},x_{m_{k}}\right)=\varepsilon . \end{array}$$

Also,

(17) G(xnk,xmk,xmk)  ≤G(xnk,xmk+1,xmk+1)+G(xmk+1,xmk,xmk)  ≤G(xnk,xmk+1,xmk+1)+G(xmk,xmk+1,xmk+1)   +G(xmk,xmk+1,xmk+1)

implies that *ε* ≤ lim⁡_*k*→*∞*_⁡*G*(*x*_*n*_*k*__, *x*_*m*_*k*_+1_, *x*_*m*_*k*_+1_).

On the other hand,

(18) G(xnk,xmk+1,xmk+1)  ≤G(xnk,xmk,xmk)+G(xmk,xmk+1,xmk+1)

combined with (13) and (16) results in lim⁡_*k*→*∞*_⁡*G*(*x*_*n*_*k*__, *x*_*m*_*k*_+1_, *x*_*m*_*k*_+1_) ≤ *ε* so that

(19) $$\begin{array}{c} \underset{k\to \infty }{\lim}G\left(x_{n_{k}},x_{m_{k}+1},x_{m_{k}+1}\right)=\varepsilon . \end{array}$$

Now,

(20) G(xnk,xmk,xmk)  ≤G(xnk,xmk+2,xmk+2)+G(xmk+2,xmk,xmk)  ≤G(xnk,xmk+2,xmk+2)+G(xmk,xmk+1,xmk+1)   +G(xmk,xmk+1,xmk+2)  ≤G(xnk,xmk+2,xmk+2)+G(xmk,xmk+1,xmk+1)   +G(xmk,xmk+1,xmk+1)+G(xmk+1,xmk+1,xmk+2)  ≤G(xnk,xmk+2,xmk+2)+G(xmk,xmk+1,xmk+1)   +G(xmk,xmk+1,xmk+1)+G(xmk+1,xmk+2,xmk+2)   +G(xmk+1,xmk+2,xmk+2)

gives that *ε* ≤ lim⁡_*k*→*∞*_⁡*G*(*x*_*n*_*k*__, *x*_*m*_*k*_+2_, *x*_*m*_*k*_+2_), and

(21) G(xnk,xmk+2,xmk+2)  ≤G(xnk,xmk,xmk)+G(xmk,xmk+2,xmk+2)  ≤G(xnk,xmk,xmk)+G(xmk,xmk+1,xmk+1)   +G(xmk+1,xmk+2,xmk+2)

implies by (13) and (19) that

(22) $$\begin{array}{c} \underset{k\to \infty }{\lim}G\left(x_{n_{k}},x_{m_{k}+2},x_{m_{k}+2}\right)\leq \varepsilon . \end{array}$$

Hence,

(23) $$\begin{array}{c} \underset{k\to \infty }{\lim}G\left(x_{n_{k}},x_{m_{k}+2},x_{m_{k}+2}\right)=\varepsilon . \end{array}$$

Also, from (16) and

(24) G(xnk,xmk,xmk)  ≤G(xnk,xmk,xmk+1)+G(xmk,xmk+1,xmk+1)

we obtain *ε* ≤ lim⁡_*k*→*∞*_⁡*G*(*x*_*n*_*k*__, *x*_*m*_*k*__, *x*_*m*_*k*_+1_).

But from

(25) G(xnk,xmk,xmk+1)  ≤G(xnk,xmk,xmk)+G(xmk,xmk,xmk+1)  ≤G(xnk,xmk,xmk)+G(xmk,xmk+1,xmk+1)   +G(xmk,xmk+1,xmk+1)

together with (13) and (16), we get lim⁡_*k*→*∞*_⁡*G*(*x*_*n*_*k*__, *x*_*m*_*k*__, *x*_*m*_*k*_+1_) ≤ *ε*. Thus,

(26) lim⁡k→∞⁡G(xnk,xmk,xmk+1)=ε,G(xnk,xmk+1,xmk+1)  ≤M(xnk,xmk+1,xmk+1)  =max⁡{G(xnk,xmk+1,xmk+1),G(xnk,fxnk,fxnk),G(xmk+1,fxmk+1,fxmk+1),[G(xnk,fxmk+1,fxmk+1)+G(xmk+1,fxnk,fxnk)]×(2)−1}  =max⁡{G(xnk,xmk+1,xmk+1),G(xnk,xnk+1,xnk+1),G(xmk+1,xmk+2,xmk+2),[G(xnk,xmk+2,xmk+2)+G(xmk+1,xnk+1,xnk+1)]×(2)−1}  ≤max⁡{G(xnk,xmk+1,xmk+1),G(xnk,xnk+1,xnk+1),G(xmk+1,xmk+2,xmk+2),[G(xnk,xmk+2,xmk+2)+G(xnk,xnk+1,xnk+1)+G(xnk,xmk,xmk+1)]×(2)−1}.

This gives

(27) ε≤lim⁡k→∞M(xnk,xmk+1,xmk+1)≤max⁡{ε,0,0,[ε+ε]2}=ε

and so

(28) $$\begin{array}{c} \underset{k\to \infty }{\lim}M\left(x_{n_{k}},x_{m_{k}+1},x_{m_{k}+1}\right)=\varepsilon . \end{array}$$

Also,

(29) $$\begin{array}{c} \underset{k\to \infty }{\lim}N\left(x_{n_{k}},x_{m_{k}+1},x_{m_{k}+1}\right)=\left(\varepsilon ,0,0,\varepsilon ,\varepsilon \right). \end{array}$$

From (4), we obtain

(30) ψ(G(xnk+1,xmk+2,xmk+2))=ψ(G(fxnk,fxmk+1,fxmk+1))≤ψ(M(xnk,xmk+1,xmk+1)) −φ(N(xnk,xmk+1,xmk+1)),

which on taking the upper limit as *k* → *∞* implies that

(31) $$\begin{array}{c} \psi \left(\varepsilon \right)\leq \psi \left(\varepsilon \right)-\varphi \left(\varepsilon ,0,0,\varepsilon ,\varepsilon \right), \end{array}$$

a contradiction as  *ε* > 0.

It follows that {*x*_*n*_} is a *G*-Cauchy sequence and by *G*-completeness of *X*, there exists *u* ∈ *X* such that {*x*_*n*_}*G*-converges to *u* as *n* → *∞*. If *f* is continuous, then it is clear that *fu* = *u*. Next, if *X* has a sequential limit comparison property, then we have *x*_*n*_⪯*u* for all *n* ∈ *ℕ*. From (4), we have

(32) ψ(G(xn+1,fu,fu))  =ψ(G(fxn,fu,fu))  ≤ψ(M(xn,u,u))−φ(N(xn,u,u)),

where

(33) M(xn,u,u)  =max⁡{G(xn,u,u),G(xn,fxn,fxn),G(u,fu,fu),[G(xn,fu,fu)+G(u,fxn,fxn)]2}  =max⁡{G(xn,u,u),G(xn,xn+1,xn+1),G(u,fu,fu),[G(xn,fu,fu)+G(u,xn+1,xn+1)]2},N(xn,u,u)  =(G(xn,u,u),G(xn,fxn,fxn),G(u,fu,fu),G(xn,fu,fu),G(u,fxn,fxn))  =(G(xn,u,u),G(xn,xn+1,xn+1),G(u,fu,fu),G(xn,fu,fu),G(u,xn+1,xn+1)).

This implies that lim⁡_*n*→*∞*_⁡*M*(*x*_*n*_, *u*, *u*) = *G*(*u*, *fu*, *fu*). Thus, from (32), we obtain

(34) ψ(G(u,fu,fu))=lim⁡ sup⁡n→∞ψ(G(fxn,fu,fu))≤lim⁡ sup⁡n→∞[ψ(M(xn,u,u))−φ(M(xn,u,u))]≤ψ(G(u,fu,fu)) −φ(0,0,G(u,fu,fu),G(u,fu,fu),0).

This gives  *φ*(0,0, *G*(*u*, *fu*, *fu*), *G*(*u*, *fu*, *fu*), 0) = 0  so that  *G*(*u*, *fu*, *fu*) = 0  and, hence, *fu* = *u*.

**Corollary 10**

Let  (*X*, ⪯)  be a partially ordered set and  *f*  be a nondecreasing self mapping on a complete *G*-metric space  *X*  satisfying

(35) $$\begin{array}{c} \psi \left(G\left(fx,fy,fy\right)\right)\leq \psi \left(M\left(x,y,y\right)\right)-\varphi \left(N\left(x,y,y\right)\right), \end{array}$$

where *ψ* ∈ Ψ, *φ* ∈ Φ with

(36) M(x,y,y)=a1G(x,y,y)+a2G(x,fx,fx) +a3G(y,fy,fy),a4[G(x,fy,fy)+G(y,fx,fx)],N(x,y,y)=(G(x,y,y),G(x,fx,fx),G(y,fy,fy),G(x,fy,fy),G(y,fx,fx))

for all *x*, *y* ∈ *X* with *x*⪯*y* with *a*_*i*_ ≥ 0 for all *i* = 1,2, 3,4 with *a*_1_ + *a*_2_ + *a*_3_ + 2*a*_4_ ≤ 1. Suppose that there exists *x*_0_ ∈ *X* with *x*_0_⪯*fx*_0_. If *f* is continuous or *X* has a sequential limit comparison property, then *f* has a fixed point in *X*.

Now we give an example to illustrate above result.

**Example 11**

Let *X* = [0,1] and *G*(*x*, *y*, *z*) = max⁡{|*x* − *y*|, |*y* − *z*|, |*z* − *x*|} be a *G*-metric on *X*. Define *f* : *X* → *X* by

(37) $$\begin{array}{c} f\left(x\right)=\frac{x}{12}\;\forall x\in X. \end{array}$$

We take *ψ*(*t*) = (3/4)*t* and *φ*(*t*_1_, *t*_2_, *t*_3_, *t*_4_, *t*_5_) = (1/12)(*t*_1_ + *t*_2_ + *t*_3_ + *t*_4_ + *t*_5_) for all *t*, *t*_1_, *t*_2_, *t*_3_, *t*_4_, *t*_5_ ∈ [0, *∞*).

Now, for all *x*, *y* ∈ *X* with *x*⪯*y*, we have

(38) $$\begin{array}{c} G\left(x,fx,fx\right)=\frac{11x}{12},\;\;G\left(y,fy,fy\right)=\frac{11y}{12},\\ \frac{\left[G\left(x,fy,fy\right)+G\left(y,fx,fx\right)\right]}{2}=\frac{\left|12x-y\right|+12y-x}{24}. \end{array}$$

So that

(39) G(fx,fy,fy)=112(y−x)≤34[16(y−x)+16(11x12)+16(11y12)+16(|12x−y|+12y−x24)] −112[(y−x)+11x12+11y12+|12x−y|+12y−x24]=34M(x,y,y)−112N(x,y,y)=ψ(M(x,y,y))−φ(N(x,y,y)).

Thus, (35) is satisfied with *a*_1_ = *a*_2_ = *a*_3_ = *a*_4_ = 1/6, where *a*_1_ + *a*_2_ + *a*_3_ + 2*a*_4_ ≤ 1. Hence, the conditions of Corollary 10 are satisfied and 0 is the fixed point of *f*.

**Corollary 12**

Let (*X*, ⪯) be a partially ordered set and *f* be a nondecreasing self mapping on a complete *G*-metric space *X* satisfying

(40) $$\begin{array}{c} G\left(fx,fy,fy\right)\leq M\left(x,y,y\right)-\varphi \left(M\left(x,y,y\right)\right), \end{array}$$

where  *φ* ∈ Φ,

(41) M(x,y,y)=max⁡{G(x,y,y),G(x,fx,fx),G(y,fy,fy),[G(x,fy,fy)+G(y,fx,fx)]2},N(x,y,y)=(G(x,y,y),G(x,fx,fx),G(y,fy,fy),G(x,fy,fy),G(y,fx,fx))

for all *x*, *y* ∈ *X* with *x*⪯*y*. Suppose that there exists *x*_0_ ∈ *X* with *x*_0_⪯*fx*_0_. If *f* is continuous or *X* has a sequential limit comparison property, then *f* has a fixed point in *X*.

**Corollary 13**

Let (*X*, ⪯) be a partially ordered set and *f* be a nondecreasing self mapping on a complete *G*-metric space *X* satisfying

(42) G(fx,fy,fy)≤M(x,y,y)−φ(N(x,y,y)),

where *φ* ∈ Φ,

(43) M(x,y,y)=a1G(x,y,y)+a2G(x,fx,fx) +a3G(y,fy,fy)+a4[G(x,fy,fy)+G(y,fx,fx)],N(x,y,y)=(G(x,y,y),G(x,fx,fx),G(y,fy,fy),G(x,fy,fy),G(y,fx,fx))

for all *x*, *y* ∈ *X* with *x*⪯*y*, where *a*_*i*_ > 0 for *i* = 1,2, 3,4 with *a*_1_ + *a*_2_ + *a*_3_ + 2*a*_4_ ≤ 1. Suppose that there exists  *x*_0_ ∈ *X*  with  *x*_0_⪯*fx*_0_. If  *f*  is continuous or  *X*  has a sequential limit comparison property, then *f* has a fixed point in  *X*.
